# Transfusion and mortality after major trauma in patients on direct oral anticoagulants: a multicentre cohort study

**DOI:** 10.1186/s13049-026-01640-2

**Published:** 2026-05-30

**Authors:** Johan Henrik Wersäll, Henrik Hedelin, Alexandra Vidlund, Elin Ekdahl, Jacob Kjellberg, Peter U. Larsson, Leo Normark, Dominika Högberg

**Affiliations:** 1 Department of Anaesthesiology and Intensive Care Medicine, Sahlgrenska University Hospital, Gothenburg, Sweden; 2 Department of Anaesthesiology and Intensive Care Medicine, Institute of Clinical Sciences, Sahlgrenska Academy, University of Gothenburg, Gothenburg, Sweden; 3 Department of Orthopedics, NU Hospital Group, Trollhättan, Sweden; 4 Department of Orthopedics, Sahlgrenska Academy, Institute of Clinical Sciences, University of Gothenburg, Gothenburg, Sweden; 5 Department of Anaesthesiology, Södra Älvsborg Hospital, Borås, Sweden; 6 Department of Anaesthesiology and Intensive Care, Skaraborg Hospital, Skövde, Sweden; 7 Department of Surgery, Sahlgrenska University Hospital, Gothenburg, Sweden; 8 Department of Surgery, Sahlgrenska Academy, Institute of Clinical Sciences, University of Gothenburg, Gothenburg, Sweden

## Abstract

**Background:**

Major trauma in patients with direct oral anticoagulants (DOACs) is increasing as treatment with DOACs is becoming more prevalent. DOACs add complexity to trauma due to increased risk of bleeding. The primary aim of this study was to compare the likelihood of packed red blood cell (PRBC) transfusion and PRBC volumes in patients with DOACs to non-anticoagulated controls after major trauma. Secondary aims were to compare mortality rates between these groups.

**Methods:**

This was a multicentre retrospective cohort study with four participating hospitals. Inclusion criteria were age ≥ 18 years and admission for trauma with New Injury Severity Score (NISS) > 15 from Jan 1, 2019, to Dec 31, 2023. The Swedish Trauma Registry and medical records were used to identify patients and to retrieve data. For primary weighted analyses, propensity scores for DOACs were estimated using sex, age, injury mechanism, ASA class, antiplatelet treatment, and NISS. Non-weighted adjusted regression models were used for complementary analyses.

**Results:**

A total of 1817 patients were admitted for major trauma during the study period, of which 756 patients were included in the analytic cohort. PRBC was administered to 30.8% of patients with DOACs and to 20.7% of controls. PRBC likelihood was higher among patients with DOACs (OR 1.73, 95% CI 1.03–2.91). The number of PRBC units among patients who received transfusions was not significantly higher for DOACs (GMR 1.11, 95% CI 0.79–1.56). Mortality rates within 24 h and 30 days did not differ significantly in primary analyses (OR 1.82, 95% CI 0.78–4.23 and OR 1.34, 95% CI 0.80–2.24, respectively).

**Conclusions:**

Patients with DOACs were more likely to receive PRBC transfusion, but PRBC volumes were not significantly different among transfused patients. Mortality did not differ significantly in the primary weighted analyses.

## Introduction

Direct oral anticoagulants (DOACs) have become widely used for prevention and treatment of thromboembolic disease [1–3]. Predictable pharmacokinetics, relatively few drug interactions, and a favourable safety profile compared to warfarin have made DOACs the most commonly prescribed group of oral anticoagulants for the majority of indications requiring long-term anticoagulation. Ongoing treatment with DOACs is increasingly common among patients admitted for trauma.

Management of patients with major trauma is inherently complex, and impaired haemostasis and increased risk of bleeding due to pre-injury anticoagulant treatment add to this complexity. Although DOACs offer several advantages compared to vitamin K antagonists, they may create challenges in trauma settings, since specific reversal agents for DOACs may be unavailable or costly, and anticoagulant activity can be difficult to assess rapidly [4, 5].

Modern damage control resuscitation principles including permissive hypotension and early control of haemorrhage are applicable to anticoagulated patients with major trauma [6–8], and transfusion should be guided by haemodynamic status, evidence of bleeding, laboratory findings, and clinical context [7–11].

There is a need for studies that specifically address pre-injury treatment with DOACs in major trauma and its association with early transfusion needs and mortality. Investigating whether DOAC-treated patients differ from non-anticoagulated patients with respect to transfusions and mortality may increase understanding of the impact of this type of drugs in major trauma.

The primary aim of this study was to compare the likelihood and volumes of packed red blood cell (PRBC) transfusion in DOAC-treated patients and non-anticoagulated patients. The secondary aim was to compare 24-hour and 30-day mortality rates between these two groups in a multi-centre Swedish setting.

## Methods

### Ethics

Ethical approval for the conduct of this study (No. 2024-06778-02) was granted by the Swedish Ethical Review Authority on 4 November 2024. The Swedish Trauma Register (SweTrau) approved registry data extraction. The Western Healthcare Region provided consent to data extraction from medical records in accordance with the study protocol.

### Study design and population

The study was designed as a multicentre retrospective cohort study of adult patients admitted for major trauma to four hospitals in the Western Healthcare region in Sweden (the Sahlgrenska Hospital, the Northern Älvsborg County Hospital, the Södra Älvsborg Hospital, and the Skaraborg Hospital). These hospitals are the major trauma-receiving hospitals of this region. The study period was set to five consecutive years from January 1, 2019, to December 31, 2023. Eligible patients were those with New Injury Severity Score (NISS) > 15 (definition of major trauma), an American Society for Anaesthesiology physical status (ASA) [12] class ≥ 2, and age ≥ 18 years at hospital admission.

Eligibility was restricted to ASA class ≥ 2 to improve comparability, because DOAC treatment reflects underlying disease and is therefore not compatible with ASA class I.

Patients with pre-injury treatment with warfarin or with incomplete and irretrievable data were excluded. Missing data for the outcome variables were assumed to be missing completely at random [13]. The Swedish trauma register (SweTrau) was used to identify patients and to retrieve data on sex, age, mechanism of injury, date of trauma, NISS, 24 h- and 30-day mortality, and ASA class. Medical records were then reviewed to retrieve data on pre-injury treatment with the DOACs apixaban, dabigatran, edoxaban, and rivaroxaban; antiplatelet agents; packed red blood cells (PRBCs) transfusions; and administration of prothrombin complex concentrate (PCC). Use of the DOACs, warfarin, PCC and antiplatelet agents was identified from the medication list and by examining discharge notes. PRBCs were counted by examining discharge notes and transfusion journals.

### Endpoints

The primary endpoint was defined as likelihood of PRBC administration within the first 24 h after admission for trauma.

Secondary endpoints were number of PRBCs among transfused patients within 24 h after admission, and mortality 24 h and 30 days after admission for trauma.

### Statistical analysis

For descriptive purposes, means and standard deviations (SD) were presented for continuous variables, and counts and percentages for categorical variables. The primary estimand was the average treatment effect on the treated (ATT). As complementary analyses, adjusted regression models without weighting were used. Propensity scores for DOAC treatment were estimated by logistic regression including age, sex, ASA class, injury mechanism, antiplatelet use, and NISS.

For binary outcomes, a weighted generalized linear model with binomial distribution and logit link was used (weighted logistic regression) with robust standard errors to estimate odds ratios (ORs) comparing DOAC-treated patients with weighted controls. Event rates were presented by DOAC group and ORs with 95% confidence intervals (CIs) and p values. As a complementary analysis, a standard multivariable logistic regression was fitted to the unweighted full control cohort including DOAC and the covariates from the propensity score model to yield covariate adjusted (conditional) ORs with 95% CIs.

Number of PRBC transfusions within 24 h was analyzed using a negative binomial regression model with a log link, weighted by the ATT weights and with robust (sandwich) standard errors. Model-based geometric means were reported with 95% CIs for each DOAC group and the geometric mean ratio (GMR) with 95% CI and p value. The GMR may be interpreted as percent change in expected counts. As a complementary analysis, an unweighted negative binomial regression was fitted to the full control cohort including DOAC exposure and the same covariates used in the propensity score model to obtain covariate adjusted (conditional) GMRs with 95% CIs. Since early death within 24 h could lead to a restricted number of PRBC transfusions, ATT-weighted and conventional multivariable sensitivity analyses were performed including only patients who survived > 24 h. Moreover, to compare PRBC volume requirements among those patients who received transfusions, analyses were performed that only included transfused patients.

All analyses were performed in SAS 9.4 (SAS Institute Inc., Cary, NC, USA). All tests were two sided and *p* < 0.05 was considered statistically significant.

## Results

### Descriptive statistics (Table 1)

**Table 1:** Baseline characteristics of analytic cohort

Variable	Controls (*n* = 639)	Weighted controls	DOAC (*n* = 117)	SMD^1^
**Sex – n (%)**				
Male	456 (71.4%)	(63.3%)	75 (64.1%)	0.02
Female	183 (28.6%)	(36.7%)	42 (35.9%)	
**Age (years)**,** mean (SD)**	62.1 (19.2)	77.2 (12.2)	77.0 (10.8)	0.04
**Injury mechanism – n (%)**				
Motor vehicle accident	101 (15.8%)	(21.2%)	23 (19.7%)	
Motorcycle accident	40 (6.3%)	(4.4%)	6 (5.1%)	
Bicycle accident	41 (6.4%)	(6.4%)	9 (7.7%)	
Injured pedestrian	0 (0.0%)	(0.0%)	1 (0.9%)	
Other vehicle-related injury	0 (0.0%)	(0.0%)	1 (0.9%)	
Stab / sharp object	36 (5.6%)	(1.8%)	3 (2.6%)	
Blunt object	34 (5.3%)	(4.9%)	4 (3.4%)	
Same-level fall (low-energy)	182 (28.5%)	(39.6%)	46 (39.3%)	
Fall from height (high-energy)	205 (32.1%)	(21.7%)	24 (20.5%)	
**ASA class – n (%)**				
II	414 (64.8%)	(39.6%)	45 (38.5%)	
III	210 (32.9%)	(55.6%)	66 (56.4%)	
IV	15 (2.3%)	(4.8%)	6 (5.1%)	
**NISS**,** median (IQR)**	22 (18–34)	22 (18–29)	22 (19–27)	
**NISS**,** mean (SD)**	27.9 (12.6)	26.9 (11.5)	26.9 (11.2)	0.00
**Antiplatelet therapy – n (%)**				
No	523 (81.8%)	(94.1%)	110 (94.0%)	
Yes	116 (18.2%)	(5.9%)	7 (6.0%)	0.00

^1^SMD = standardized mean difference after propensity-score weighting. SMD < 0.1 indicates good covariate balance

**Fig. 1:**
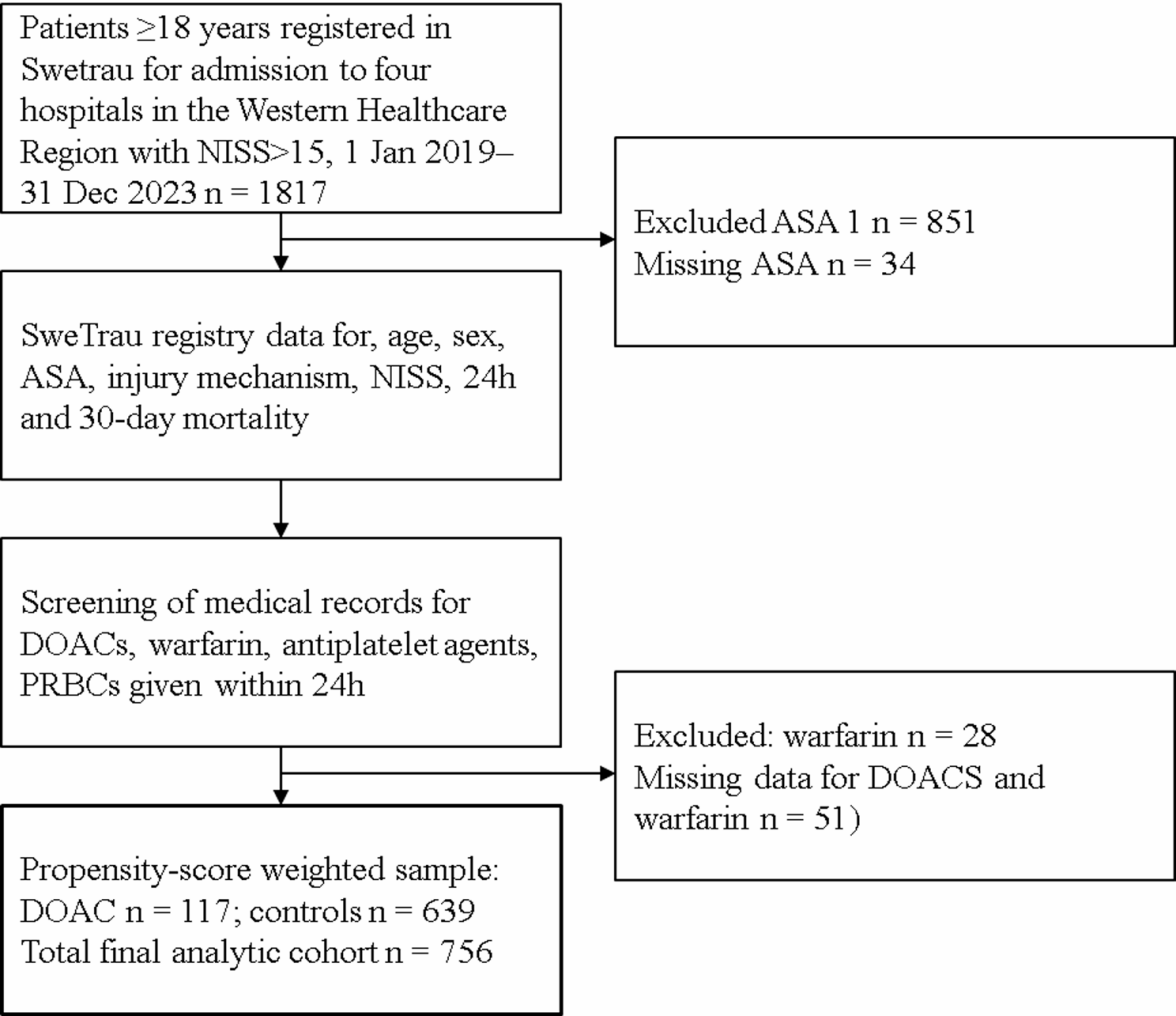
Flowchart describing inclusion and exclusion procedures in the study

Analytic cohort after inclusion and exclusion criteria were met (Fig. 1). Distributions of non-weighted and weighted non-anticoagulated controls compared to patients with DOACs. Weighting was performed for the average treatment effect of the treated (ATT) as estimand

Abbreviations: DOAC=direct oral anticoagulant. ASA=American Society of Anesthesiology physical status classification. NISS = New Injury Severity Score. IQR=Interquartile Range

In total, 1817 patients with NISS > 15 were registered in SweTrau from January 1, 2019, to December 31, 2023. Of these, 756 patients were included in the final analytical cohort (Fig. 1). The baseline characteristics of the analysis cohort and balance after weighting are shown in Table 1. DOAC-treated patients were older and had higher ASA class than controls but with similar injury severity by NISS. Compared with controls, DOAC-treated patients more commonly sustained low-energy same-level falls (39% vs. 29%) and less commonly falls from height (21% vs. 32%). Patients with DOACs had greater comorbidity, with ASA class 3–4 present in 72/117 (61.5%) of DOAC-treated patients and 225/639 (35.2%) of controls. Pre-injury antiplatelet therapy was more frequent among controls (18%) than in DOAC-treated patients (6.0%). NISS was similar between controls and DOAC-treated patients for both median and mean values. PCC was administered to 40/116 (34%) of the patients with DOACs.

After ATT weighting, covariate balance between DOAC-treated patients and weighted controls was good, as shown by small standardised mean differences in Table 1. A c statistic of 0.82 (95%CI 0.77–0.85) was obtained. Standardized mean differences comparing the DOAC group with the weighted controls were all < 0.05, indicating good balance.

### Analyses (Table 2)

**Table 2:** Analyses comparing patients with direct oral anticoagulants (DOACs) and non-anticoagulated patients (controls) after major trauma

Outcome	DOAC	Control	ATT-weighted effect	*p*	Adjusted effect	*p*
**A. Binary outcomes (odds ratios)**						
Death within 24 h	11/117 (9.4%)	37/639 (5.8%)	OR 1.82 (0.78 to 4.23)	0.16	OR 2.20 (0.91 to 5.30)	0.080
Death within 30 days	35/117 (29.9%)	113/639 (17.7%)	OR 1.34 (0.80 to 2.24)	0.27	OR 1.80 (1.04 to 3.09)	0.034
Any PRBC transfusion within 24 h	36/117 (30.8%)	132/639 (20.7%)	OR 1.73 (1.03 to 2.91)	0.038	OR 1.99 (1.19 to 3.33)	0.009
**B. Count outcomes: number of PRBC units within 24 h (geometric mean ratios)**						
*Including patients with 0 transfusions*						
Main analysis	GM 1.32 (*n* = 117)	GM 0.92 (*n* = 639)	GMR 1.77 (1.05 to 2.96)	0.031	GMR 2.43 (1.32 to 4.46)	0.004
Sensitivity: excluding deaths within 24 h	GM 1.32 (*n* = 106)	GM 0.87 (*n* = 602)	GMR 1.86 (1.07 to 3.24)	0.028	GMR 2.41 (1.28 to 4.55)	0.007
*Among transfused patients only (excluding 0 transfusions)*						
Main analysis	GM 4.28 (*n* = 36)	GM 4.45 (*n* = 132)	GMR 1.11 (0.79 to 1.56)	0.55	GMR 1.36 (0.96 to 1.94)	0.086
Sensitivity: excluding deaths within 24 h	GM 4.24 (*n* = 33)	GM 4.48 (*n* = 117)	GMR 1.08 (0.75 to 1.58)	0.67	GMR 1.28 (0.88 to 1.85)	0.19

Likelihood of packed red blood cell (PRBC) transfusion within 24 h after admission, and comparison of PRBC transfusion volumes for the full cohort and among patients who were transfused. Mortality rates are within 24 h and 30 days. Propensity-score weighting was used for estimation of the average treatment effect on the treated (ATT), and non-weighted adjusted regression was used for complementary analyses

Abbreviations: ATT=average treatment effect on the treated; CI=confidence interval; DOAC=direct oral anticoagulant; GM=Geometric Mean; GMR=geometric mean ratio; OR=odds ratio; PRBC=packed red blood cells

#### Primary endpoint: likelihood of receiving PRBC transfusion within 24 h

The proportion of patients receiving at least one unit of packed red blood cells (PRBCs) within the first 24 h after admission was higher among DOAC-treated patients than among non-anticoagulated controls (31% vs. 21%). In propensity-score ATT-weighted analyses, DOAC treatment was associated with higher likelihood of PRBC transfusion within 24 h compared to non-anticoagulated patients (OR 1.73, 95%CI 1.03–2.91, Table 2). This association remained statistically significant in a sensitivity analysis using a conventional multivariable adjusted model (OR 1.99, 95% CI 1.19–3.33).

#### Secondary endpoint: number of PRBC transfusions within 24 h

Among all patients in the final cohort (including patients who did not receive any transfusions), DOAC treatment was associated with an ATT-weighted point estimate of nearly 80% higher number of PRBC transfusions within 24 h compared with non-anticoagulated patients (GMR 1.77, 95% CI 1.05–2.96, Table 2). The positive association between DOACs and higher number of PRBC transfusions remained statistically significant in a conventional multivariable adjusted model (GMR 2.43, 95% CI 1.32–4.46).

In analyses excluding patients who died within 24 h, DOAC treatment remained associated with a higher number of PRBC transfusions, both in ATT-weighted analysis (GMR 1.86 95%CI 1.07–3.24, Table 2) and in a conventional multivariable model (GMR 2.41 95%CI 1.28–4.55).

##### PRBC transfusion volume restricted to only patients who were transfused

**Fig. 2:**
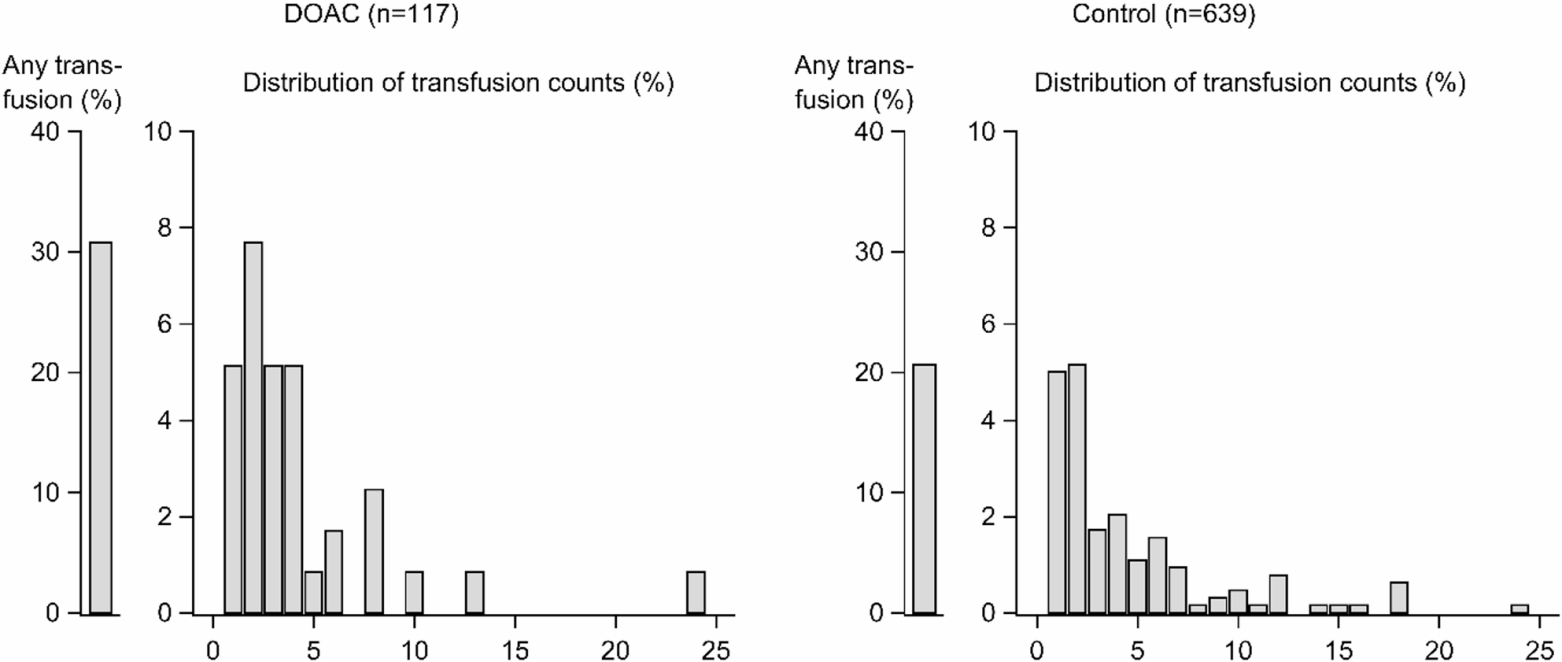
Comparison of proportions of packed red blood cells units (PRBC) between patients treated with direct oral anticoagulants (DOACs) and controls (non-anticoagulated patients) admitted for major trauma between the years 2019 to 2023. Note that the Y-axes are truncated for visual clarity

Most patients in the full analytical cohort did not receive any PRBC transfusions (Fig. 2). Among those who received transfusions, the mean PRBCs transfused for DOAC-treated patients were 4.3 units, while non-anticoagulated controls received a mean PRBC transfusion of 3.9 units. No statistically significant differences were seen in either the ATT-weighted analysis (GMR 1.11 95% CI 0.79–1.56, Table 2) or in the conventional multivariable adjusted model (GMR 1.36, 95%CI 0.96–1.94). In sensitivity analyses including only patients who survived > 24 h, the mean PRBCs transfused for DOAC-treated patients were 4.2 units, while non-anticoagulated controls received a mean PRBC transfusion of 3.9 units. No statistically significant differences were observed in these sensitivity analyses in either the ATT-weighted analysis or the conventional multivariable adjusted analysis (Table 2).

#### Secondary endpoint: 24-hour and 30-day mortality

Overall, 24-hour mortality rate was 9% (11/117) in DOAC-treated patients versus 6% (37/639) in controls. Analysis of 24-hour mortality rates between DOAC-treated and non-anticoagulated patients showed no statistically significant differences in either the ATT-weighted or the conventional multivariable adjusted model. The 30-day mortality rate was 30% (35/117) in DOAC-treated patients versus 17.7% (113/639) in controls. DOAC-treated patients had higher mortality rates within 30 days in the multivariable adjusted model while no statistically significant difference was observed in the ATT-weighted model.

## Discussion

### Transfusion

In this study of patients with major trauma, pre-injury treatment with DOACs was associated with a higher likelihood of receiving any PRBC transfusion within 24 h compared to no anticoagulant treatment. However, among transfused patients, the number of PRBC units was similar between DOAC-treated and non-anticoagulated patients. The higher overall PRBC transfusion rate in the DOAC group thus appeared to be primarily driven by a higher proportion of patients receiving any transfusion in this group, rather than by larger PRBC volumes among the DOAC-treated patients who were transfused (Table 2; Fig. 2).

There may be several explanations to these findings. The decision to initiate PRBC transfusion may have been influenced by several factors. Apart from actual haemodynamic status, factors like suspected bleeding, mechanism and types of injury, laboratory findings, and concerns about DOAC treatment may all have been triggers of transfusion. Although transfusion protocols do not include recommendations of PRBC transfusion in anticoagulated patients per se, anticipated bleeding problems in this group might have contributed to higher likelihood of a transfusion in patients with DOACs. In contrast, the PRBC volumes among transfused patients may to a greater extent reflect physiological response to resuscitation and success of haemorrhage control. The non-significant difference in transfusion volumes between the groups in this study is in line with other recent studies on patients with trauma, which also showed similar PRBC counts between patients with DOACs compared to non-anticoagulated patients [14, 15]. It is possible that similar PRBC volumes among non-anticoagulated and DOAC-treated patients may have been due to efficient haemorrhage control, achieved regardless of pre-injury anticoagulation by applying efficient damage control resuscitation until surgical haemostasis. Additionally, the extent of anticoagulant pharmacological activity may have been decreased at the time of injury, or at hospital admission among several patients in the DOAC group. Factor Xa inhibitors in Swedish clinical use (apixaban, edoxaban and rivaroxaban) have a half-life of approximately up to 12 h [16–18] and dabigatran up to 17 h [19]. The present study did not include data on timing of the last DOAC dose, DOAC plasma concentration, and anti-Xa activity and antithrombin activity, and the relationship between anticoagulant activity and transfusion volume could therefore not be determined. Studies regarding timing, dose, and anticoagulant activity are warranted to further assess the relationship between DOAC treatment and transfusion requirements after major trauma.

### Mortality

Moderately higher absolute mortality rates were seen within 24 h among DOAC-treated patients compared to controls (9% vs. 6%), similar to findings in previous studies that also did not find these difference statistically significant [20, 21].

Absolute mortality rates after 30 days were substantial (30% for vs. 18% for non-anticoagulated patients within 30 days) Although the differences in mortality did not show statistical significance within 24 h and 30 days (except for 30-day mortality in the conventional multivariable analysis), tendencies for higher risk of death among patients with DOACs were indicated by the point estimates. It is plausible that statistical power in this study could have been insufficient to detect significance under the 0.05 level.

This study has some limitations. First, an observational, retrospective design warrants interpretational caution mainly due to the risk of confounding. Weighting was performed to mitigate this issue, yet residual confounding may distort the findings.

Second, ASA pre-injury classes cover a broad range of pathologies within each class. These may not always align with respect to risk of bleeding and mortality in trauma. However, stratification into specified comorbidities would create challenges, since several comorbidities may be present within each patient, and since no clear evidence exists for mortality rates for each one of these in this setting.

Lastly, no distinction was made between traumatic brain injuries and non-traumatic brain injuries. Transfusion requirements were not assumed to depend on whether TBI was present or not, but anticoagulated patients with TBI might have had different mortality patterns in the study population compared to patients without TBI. However, mortality and expansion of intracranial hematoma after blunt head trauma have not shown to be high in patients with DOACs [22–24].

There are several strengths of this study that may be pointed out. One is its multicentre design, covering an entire administrative public healthcare region. Another strength is a relatively large patient cohort spanning five consecutive years with low number of missing data. The use of a validated high-quality trauma register [25] for inclusion of the target population is also a strength that may increase reliability of the results from this study.

## Conclusion

Within 24 h after major trauma, patients with ongoing DOAC treatment were more likely to receive a PRBC transfusion than non-anticoagulated patients. However, transfusion volumes among transfused patients did not differ between the groups. Mortality at 24 h and 30 days did not differ significantly between the groups in the primary weighted analyses.

## Data Availability

The data used in the current study are subject to restrictions under Swedish law and are not publicly available due to patient confidentiality. Data may be made available upon reasonable request and after approval from the Western Health Care Region (Region Västra Götaland), Sweden.

## Declarations

### Additional information

Preliminary data were presented as an abstract at the annual conference of the European Society of Anaesthesiology and Intensive Care (Euroanaesthesia 2025).
